# Genome Sequences of a Novel Vietnamese Bat Bunyavirus

**DOI:** 10.1128/genomeA.01366-16

**Published:** 2016-12-22

**Authors:** Bas B. Oude Munnink, My V. T. Phan, Lia van der Hoek, Paul Kellam, Matthew Cotten

**Affiliations:** a Wellcome Trust Sanger Institute, Hinxton, England; b Department of Viroscience, Erasmus Medical Center, Rotterdam, The Netherlands; c Laboratory of Experimental Virology, Academic Medical Center, University of Amsterdam, Amsterdam, The Netherlands; d Imperial College London, London, United Kingdom

## Abstract

To document the viral zoonotic risks in Vietnam, fecal samples were systematically collected from a number of mammals in southern Vietnam and subjected to agnostic deep sequencing. We describe here novel Vietnamese bunyavirus sequences detected in bat feces. The complete L and S segments from 14 viruses were determined.

## GENOME ANNOUNCEMENT

The *Bunyaviridae* is a diverse viral family comprising five genera. Some members are notorious for their zoonotic potential (hantavirus and Rift Valley fever virus), one can cause severe problems in cattle (Smallenberg virus), and another infects plants (tomato spotted wilt virus). Members of the enveloped *Bunyaviridae* typically enclose a segmented negative-sense single-stranded RNA genome, with the L segment encoding an RNA-dependent RNA polymerase (RdRp), the M segment encoding glycoproteins, and the S segment encoding the nucleoprotein. The combined genomic length of the three segments is 11 to 19 kb (1).

We searched for novel members of the *Bunyaviridae* in 135 bat fecal samples collected from roosting sites using an agnostic deep-sequencing approach (2). Fecal samples were processed as previously described (3), followed by sequencing on an Illumina HiSeq platform yielding 3 to 4 million 250-nucleotide (nt) paired-end reads per sample, which were *de novo* assembled using SPAdes version 3.5.0 (4), followed by improve_assembly (5). The resulting reads were subjected to a modified protein blast search using usearch (6) to identify *Bunyaviridae*-related sequences.

Fourteen of 135 samples (10%) yielded sequences with 51% amino acid identity to a small part of the RdRp of a *Rhinolophus pearsoni* bunyavirus. The M and S segments of this new Vietnamese bat bunyavirus could not be identified using simple homology searching. Therefore, Uclust (6) was used to cluster all consensus sequences of the bunyavirus-positive samples. Contigs present in over 70% of the samples were submitted to a conserved domain search (7), which yielded a putative S segment of the novel bunyavirus showing similarities to a conserved tenuivirus/phlebovirus nucleocapsid protein domain; however, no amino acid identity to known bunyaviruses could be identified. The genome lengths of the L segment of the novel Vietnamese bat bunyaviruses were 6,484 to 6,713 nucleotides (average sequence coverage, 78- to 2,619-fold). The nucleotide sequence of the L segment of the 14 isolates differed at 21 to 124 positions (98% to 100% nucleotide identity), while the S segments differed at 5 to 54 positions (97% to 100% nucleotide identity). The genome length of the S segment varied between 1,464 and 1,578 nucleotides (average sequence coverage, 47- to 849-fold).

Consistent with other studies (8, 9), no contigs with similarities to the *Bunyaviridae* M segment could be found. Either the M segments exists in these samples with greater sequence divergence precluding identification, or these viruses exist without a standard M segment, perhaps by complementation with functions from other coinfecting viruses.

In conclusion, we present the L and S genome segments of a novel Vietnamese bunyavirus. This novel virus was identified in 14 bat fecal samples, and for all viruses, the complete genome sequences of the L and S segments were determined. The lengths of the two segments of this novel unclassified bunyavirus are consistent with other members of *Phlebovirus* and the* Hantavirus* (1); however, additional research is needed to accurately classify this novel bunyavirus and resolve the M segment mystery.

### Accession number(s).

The complete genome sequences of the Vietnamese bat bunyaviruses are deposited in GenBank under the accession numbers KX886759 to KX886786.
